# Building a PPE Monitor Team as Part of a Comprehensive COVID-19 Prevention Strategy

**DOI:** 10.1017/ash.2021.88

**Published:** 2021-07-29

**Authors:** Shelley Summerlin-Long, Brooke Brewer, Amy Selimos, Mark Buchanan, Christa Clark, Karen Croyle, Cynthia Culbreth, Pam Del Monte, Lauren DiBiase, Lori Hendrickson, Pam Miller, Natalie Schnell, Katherine Schultz, Lisa Stancill, Lisa Teal, David Weber, Emily Sickbert-Bennett

## Abstract

**Background:** The use of personal protective equipment (PPE) is a critical intervention in preventing the spread of transmission-based infections in healthcare settings. However, contamination of the skin and clothing of healthcare personnel (HCP) frequently occurs during the doffing of PPE. In fact, nearly 40% of HCP make errors while doffing their PPE, causing them to contaminate themselves. PPE monitors are staff that help to promote their colleagues’ safety by guiding them through the PPE donning and doffing processes. With the advent of the COVID-19 pandemic in early 2020, the UNC Medical Center chose to incorporate PPE monitors as part of its comprehensive COVID-19 prevention strategy, using them in inpatient areas (including COVID-19 containment units and all other units with known or suspected SARS-CoV-2–positive patients), procedural areas, and outpatient clinics. **Methods:** Infection prevention and nursing developed a PPE monitoring team using redeployed staff from outpatient clinics and inpatient areas temporarily closed because of the pandemic. Employee training took place online and included fundamentals of disease transmission, hand hygiene basics, COVID-19 policies and signage, and videos on proper donning and doffing, including coaching tips. The monitors’ first shifts were supervised by experienced monitors to continue in-place training. Employees had competency sheets signed off by a supervisor. **Results:** The Medical Center’s nursing house supervisors took over management and deployment of the PPE monitoring team, and infection prevention staff continued to train new members. Eventually, as closed clinics and areas reopened and these PPE monitors returned to their regular positions, areas used their own staff to perform the role of PPE monitor. In the fall of 2020, a facility-wide survey was sent to all inpatient staff to assess their perceptions of the Medical Center’s efforts to protect them from acquiring COVID-19. It included a question asking how much staff agreed or disagreed that PPE monitors “play an important role in keeping our staff who care for COVID-19 patients safe.” Of the 626 staff who answered this question, 67.6% agreed or strongly agreed that PPE monitors played an important role in keeping staff safe. Thus far, there has been no direct transmission or clusters of COVID-19 involving HCP in COVID-19 containment units with PPE monitors. **Conclusions:** PPE monitors are an important part of a comprehensive COVID-19 prevention strategy. In early 2021, the UNC Medical Center posted and hired paid PPE monitor positions to continue this critical work in a sustainable way.

**Funding:** No

**Disclosures:** None

